# Emodin protects H9c2 cells from hypoxia-induced injury by up-regulating miR-138 expression

**DOI:** 10.1590/1414-431X20187994

**Published:** 2019-02-25

**Authors:** Xuezhi Zhang, Qiaoji Qin, Hongyan Dai, Shanglang Cai, Changyong Zhou, Jun Guan

**Affiliations:** 1 Department of Emergency Internal Medicine, The Affiliated Hospital of Qingdao University, Qingdao, China; 2 Department of Cardiology, Qingdao Municipal Hospital, Qingdao, China; 3 Department of Cardiology, The Affiliated Hospital of Qingdao University, Qingdao, China

**Keywords:** Emodin, Myocardial infarction, Hypoxia injury, miR-138, Sirt1/AKT pathway, Wnt/β-catenin pathway

## Abstract

Myocardial infarction (MI) is a common presentation for ischemic heart disease, which is a leading cause of death. Emodin is a Chinese herbal anthraquinone used in several diseases. However, the effect of emodin in hypoxia-induced injury in cardiomyocytes has not been clearly elucidated. Our study aimed to clarify the functions of emodin in hypoxia-induced injury in rat cardiomyocytes H9c2 and explore the underlying mechanism. The effects of emodin on cell viability and apoptosis were analyzed by the Cell counting kit-8 assay and flow cytometry assay, respectively. The cell proliferation- and cell apoptosis-related proteins were detected by western blot. qRT-PCR was used to determine the relative expression of miR-138. Cell transfection was performed to alter miR-138 and MLK3 expression. miR-138 target was performed by dual luciferase activity assay. Sirt1/AKT and Wnt/β-catenin pathways-related factors phosphorylation were analyzed by western blot. Emodin inhibited hypoxia-induced injury in H9c2 cells by promoting cell viability and reducing cell apoptosis. miR-138 was down-regulated by hypoxia treatment but up-regulated by emodin. Up-regulation of miR-138 alleviated hypoxia-induced cell injury. Down-regulation of miR-138 attenuated the growth-promoting effect of emodin on hypoxia-induced injury, whereas up-regulation of miR-138 enhanced the growth-promoting effects of emodin. The underlying mechanism might be by inactivating Sirt1/AKT and Wnt/β-catenin pathways. MLK3 was negatively regulated by miR-138 expression and inactivated Sirt1/AKT and Wnt/β-catenin pathways. Emodin alleviated hypoxia-induced injury in H9c2 cells via up-regulation of miR-138 modulated by MLK3, as well as by activating Sirt1/AKT and Wnt/β-catenin pathways.

## Introduction

Myocardial infarction (MI) is a common presentation for ischemic heart disease and coronary artery disease, which is a leading cause of death among all cardiovascular diseases (1). The classification differentiates between type 1 MI, due to thrombosis of an atherosclerotic plaque, and type 2 MI, due to the imbalanced supply and demand of myocardial blood that might be triggered by many acute medical and surgical conditions (2). The immediate revascularization and optimal medical therapy may improve prognosis in these patients. In spite of advances of medical and interventional treatment, mortality in patients with acute MI remains high (3). Therefore, new medicines and new therapies are urgently needed.

Emodin (1,3,8-trihydroxy-6-methyl-9,10-anthracenedione, C_15_H_10_O_5_) is a Chinese herbal anthraquinone isolated from some traditional Chinese herbs including Rhubarb and *Polygonum cuspidatum*. Emodin exhibits numerous biological activities, such as anti-tumor (4), anti-viral (5), anti-bacterial (6), anti-inflammation (7), and anti-oxidation (7). It was reported that emodin had potential effects to attenuate lipopolysaccharide (LPS) and hypoxia/reoxygenation-induced intestinal epithelial barrier dysfunction (8). Aloe-emodin could suppress hypoxia-induced retinal angiogenesis by inhibition of the hypoxia-inducible factor (HIF-1α)/vascular endothelial growth factor (VEGF) pathway (9). However, limited studies were performed to investigate the role of emodin in MI and other cardiovascular diseases.

microRNAs (miRNAs), a class of small noncoding RNAs, can negatively regulate gene expression at the post-transcriptional level (10). miRNAs play a pivotal role in drug efficacy and toxicity and have potential clinical implications for personalized medicine by regulating the expression of pharmacogenomic-related genes (11). A variety of miRNAs were found to modulate the efficacy of emodin, such as miR-126 (12), miR-34a (13), and miR-199a (14). Among these identified miRNAs, miR-138 was reported to protect cardiomyocytes against hypoxia-induced injury and considered an important regulator in cellular signaling pathways (15). Importantly, miR-138 has proven to be crucial in cardiac cell fate decisions including cardiac conduction, regulation of cardiac patterning, management of angiogenesis, and protection against ischemia (16). Whether emodin regulates miR-138 expression and affects hypoxia-induced injury of cardiomyocytes by regulating miR-138 expression have not been well studied. Therefore, our study aimed to explore the effect of emodin on hypoxia-induced injury in H9c2 cells and in which process the role of miR-138 regulated by emodin was investigated.

Wnt/β-catenin and sirtuin 1 (Sirt1)/protein kinase B (AKT) signaling pathway regulated a broad range of cell processes, such as cell proliferation, apoptosis, invasion, and differentiation (17,18). Meanwhile, miR-138 was observed to have close correlation with Sirt1/AKT and Wnt/β-catenin signal pathways. For example, miR-138-5p promoted tumor necrosis factor-alpha (TNF-α)-induced apoptosis in human intervertebral disc degeneration by targeting Sirt1 (19). In addition, TUG1-miR-138-5p- Sirt1-Wnt/β-catenin signaling pathway axis provided new light on the diagnosis and treatment for cervical cancer patients (20). Therefore, in our study, the potential regulation between miR-138 and these signal pathways was also explored.

## Material and Methods

### Cell culture and treatment

Rat cardiomyocytes cell line (H9c2), purchased from the American Type Culture Collection (ATCC, USA), was seeded into flasks (1×10^4^ cells/mL) in Dulbecco's modified Eagle medium (DMEM, Gibco, USA) containing 10% (v/v) fetal bovine serum (FBS, Life Science, USA), 100 U/mL penicillin, and 100 μg/mL streptomycin and incubated at 37°C under humid conditions in an atmosphere of 95% air and 5% CO_2_. H9c2 cells were incubated in a hypoxic incubator containing 1% O_2_, 94% N_2_, and 5% CO_2_ for inducing hypoxia injury. Firstly, the H9c2 cells were exposed in a hypoxia environment for 4, 8, 16, 24, and 48 h for choosing a useful hypoxia treatment time for the following experiments. At the end of each time interval for hypoxia treatment, the incubator was refilled with O_2_ to the normal condition for reoxygenation.

Emodin (Sigma-Aldrich, USA (ref: E7881, ≥90% (HPLC)) was dissolved in dimethyl sulfoxide (DMSO, Sigma Aldrich) at concentration of 100 mM and was used to treat cells at concentrations ranging from 5 μM to 20 μM.

### Cell counting kit-8 (CCK-8) assay

H9c2 cells were seeded in a 96-well plate with 5000 cells/well. After treatment, 20 μL CCK-8 solution (Beyotime, China) was added to the culture medium, and the 96-well plate was incubated for 1 h at 37°C in humidified 95% air and 5% CO_2_. The absorbance was measured at 450 nm using a Microplate Reader (Bio-Rad, USA).

### Apoptosis assay

The apoptotic cell rate was analyzed by annexin V-fluorescein isothiocyanate (FITC)/propidium iodide (PI) apoptosis detection kit (Beijing Biosea Biotechnology, China) combined with flow cytometry analysis. Cells were rinsed in PBS and then stained in PI and FITC-annexin V solution in the presence of 50 μg/mL RNase A (Sigma-Aldrich). After incubation for 2 h at room temperature in the dark, apoptotic cell rate was determined using a FACS can (Beckman Coulter, USA). The data were analyzed using FlowJo software (TreeStar, USA).

### miRNA silencing and overexpression

miR-138 inhibitor, miR-138 mimic, pc-MLK3, and the negative control (NC) were synthesized by GenePharma Co. (China). The sequence of rno miR-138 inhibitor was: 5′-CGGCCUGAUUCACAACACCAGCU-3′; the sequence of rno miR-138 mimic was: 5′-AGCUGGUGUUGUGAAUCAGGCCG-3′; the sequence of rno NC was: 5′-UCACAACCUCCUAGAAAGAGUAGA-3′. In addition, the concentration of miR-138 inhibitor, miR-138 mimic, and NC was 150, 50, and 50 nM, respectively. Cell transfection was conducted using Lipofectamine 3000 reagent (Invitrogen, USA) following the manufacturer’s instructions. In brief, 2 × 10^4^ H9c2 cells in a 24-well plate were transfected with indicated plasmid DNA, miRNA mimic 50 nM (GenePharma), miRNA inhibitor 150 nM (GenePharma), and NC 50 nM. Cells were collected 24-48 h after transfection for assay.

### Quantitative real time polymerase chain reaction (qRT-PCR) analysis

Total RNA was extracted from H9c2 cells by Trizol reagent (Invitrogen). For miR-138 detection, reverse-transcribed complementary DNA was synthesized with the PrimeScript RT reagent Kit (TaKaRa, China), and qRT-PCR was performed with SYBR Premix ExTaq (TaKaRa) with the Stratagene Mx3000P real-time PCR system (Agilent Technologies, Inc., USA). The sequence of miR-138 primer was: forward 5′-GCCGCAGCTGGTGTTGTGAAT-3′, reverse 5′-GCGAGCACAGAATTAATACGAC-3′. The sequence of U6 was: forward 5′-CTCGCTTCGGCAGCACA-3′, reverse 5′-GCGAGCACAGAATTAATACGAC-3′, and the concentration of primers was 0.2 μmol/L. The relative expression ratio of miR-138 was calculated by the 2^−ΔΔCT^ method (21). The reaction parameters were incubation at 95°C for 10 min, then 40 cycles of 95°C for 15 s, 60°C for 1 min. The threshold cycle (Ct) is defined as the cycle number at which the fluorescence passed a pre-determined threshold. For expression analysis, the experiment was designed to use the matched non-hypoxia cell as the control, so the relative quantification of miR-138 in hypoxia-treated cells was calculated using the equation: amount of target = 2^−ΔΔCt^, ΔΔCt = (CtmiR-138 - CtU6)_hypoxia_ - (CtmiR-138-CtU6)_matched non-hypoxia_. For the matched non-hypoxia cell control sample, ΔΔCt is zero and 2-ΔΔCt is 1. Melting curves were generated and 8% PAGE electrophoresis was performed for each real-time PCR to verify the amplification of only the desired product. The same method of analysis was used for the group treated with hypoxia and emodin. U6 was the internal control for miR-138 and β-actin was the internal control for mixed lineage kinase 3 (MLK3).

### Western blot

Cells were collected and lysed in RIPA lysis buffer (Beyotime) supplemented with protease inhibitors (Roche, Switzerland). The equivalent amounts of proteins (20 µg) were denatured at 100°C in loading buffer for 15 min, resolved on 8–12% sodium dodecyl sulfate-polyacrylamide gel electrophoresis (SDS-PAGE) gels, and transferred to polyvinylidene fluoride (PVDF) membranes by voltage gradient transfer. The blots were blocked overnight with 5% non-fat milk. The membrane was incubated with primary antibodies and proper secondary antibodies. The used primary antibodies against p53 (ab131442), p21 (ab109199), cyclin D1 (ab134175), caspase-3 (ab4051), cleaved-caspase-3 (ab49822), caspase-9 (ab202068), cleaved-caspase-9 (ab2324), MLK3 (ab51068), Sirt1 (ab110304), AKT (ab185633), p-AKT (ab131443), Wnt3a (ab28472), β-catenin (ab32572), and β-actin (ab8227) purchased from Abcam (UK) were used at the dilution of 1:1000. β-actin was used as a loading control. After washing, the binding antibody was visualized using the enhanced chemiluminescence assay kit (Tiangen Biotechnology Corp., China) according to the manufacturer’s instructions.

### Dual luciferase activity assay

The 3′UTR of MLK3 was generated by PCR and the luciferase reporter constructs with the miR-138 3′UTR carrying a putative miR-138-binding site into pMiR-report vector were amplified by PCR. Mutagenesis was performed when the seed region was mutated to remove all complementarity to nucleotides of miR-138. The sequence of miR-138 was 5′-GCCGGACUAAGUGUUGUGGUCGA-3′, the sequence of wild type of MLK3 was: 5′-GGGGAAAGGGGCUGACCUCAGGUGUCACCAGCACUUUU-3′, the sequence of mutant MLK3 was: 5′-GGGGAAAGGGGCUGACCUCAGGUGUGUUUGAAACUUUU-3′. Cells were co-transfected with the reporter construct, control vector, and miR-138 mimic or scramble using Lipofectamine 3000 (Life Technologies, USA). Reporter assays were done using the dual-luciferase assay system (Promega, USA) following the manufacturer’s information. In brief, the firefly luciferase reporter assay is initiated by mixing lysate with Luciferase assay reagent II (LAR II). Upon completion of the firefly luciferase assay, the firefly luminescence is quenched and Renilla luminescence is simultaneously activated by adding Stop & Glo TM Reagent to the sample tube. The value of luciferase activity was measured. The results are reported as a ratio of firefly luciferase (Fluc) activity to Renilla luciferase (Rluc) activity.

### Statistical analysis

All data are reported as means±SD from three to six samples. Data analysis was performed using GraphPad Prism version 6.0 software (GraphPad Software, USA). Student's *t-*test, one-way analysis of variance, and two-way analysis of variance were performed according to the data characteristics. P values <0.05 were considered statistically significant.

## Results

### Hypoxia induced H9c2 cell injury

**Figure 1:**
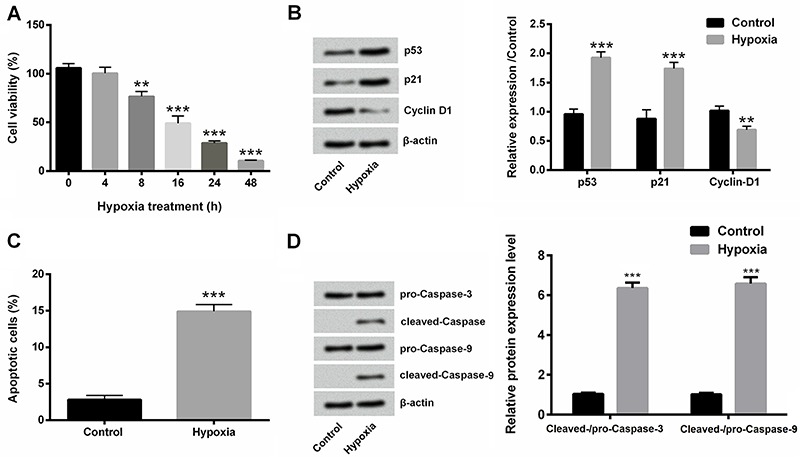
Hypoxia induced injury in H9c2 cells. *A*, Hypoxia inhibited viability detected by CCK-8 assay. *B*, Hypoxia (16 h) increased anti-proliferation protein expression and decreased pro-proliferation protein expression examined by western blot. *C*, Hypoxia (16 h) increased apoptotic cell rate measured by flow cytometry. *D*, Hypoxia (16 h) activated pro-apoptosis protein expression detected by western blot. Data are reported as means±SD of triplicates. Each experiment was performed three times. **P<0.01, ***P<0.001 compared to control (Student's *t*-test and one-way analysis of variance).

H9c2 cells were incubated in medium under the hypoxia condition for different time spans (0, 4, 8, 16, 24, and 48 h) and then cell viability was determined. The data showed that cell viability was inhibited by hypoxia treatment and the injury was enhanced with extending treatment time (P<0.01 or P<0.001, Figure 1A). We found that when the hypoxia treatment time was ≥16 h, cell viability was severely decreased. Therefore, in the following experiment, hypoxia treatment time was set as 16 h. The protein accumulated level showed that p53 rose by 2 times and p21 accumulated level rose by 1.9 times (P<0.001), and cyclin D1 reduced by 0.67 times (P<0.01, Figure 1B). Additionally, hypoxia treatment induced cell apoptosis (P<0.001, Figure 1C) and increased the ratio of cleaved-/pro-caspases-3 by 6.1 times and the ratio of cleaved-/pro-caspases-9 by 6.4 times detected by western blot (P<0.001, Figure 1D). The data suggested that hypoxia induced injury in H9c2 cells.

### Emodin inhibited hypoxia injury in H9c2 cells

**Figure 2:**
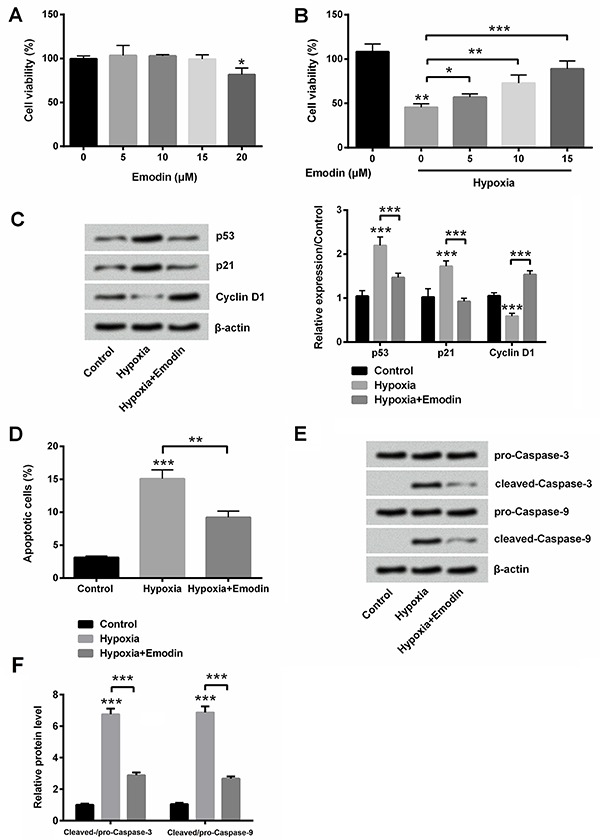
The appropriate concentration of emodin reduced the damaging effect of oxygen-deprivation on H9c2 cells. *A*, Emodin with concentration less than 20 μM had no inhibitory effect on viability of H9c2 cells detected by CCK-8 assay. *B*, Emodin promoted viability of oxygen-deprived H9c2 cells in a dose-dependent manner detected by CCK-8 assay. Emodin treatment (*C*) increased the pro-proliferation protein expression and decreased the anti-proliferation protein, (*D*) declined apoptotic cell rate, and (*E*–*F*) inhibited the pro-apoptosis protein expression. The expression of protein level was detected by western blot and cell apoptosis was measured by flow cytometry. Data are reported as means±SD of triplicates. Each experiment was performed three times. *P<0.05, **P<0.01, ***P<0.001 compared to control or as indicated (one-way analysis of variance).

Emodin exhibited no negative effect on viability of H9c2 cells at concentrations of 5, 10, and 15 μM, but cell viability was decreased by 0.82 times by emodin at the concentration of 20 μM compared with control (Figure 2A), indicating that the safe concentrations for the study should be less than 20 μM. The viability of hypoxia-stimulated H9c2 cells was increased by emodin treatment in a dose-dependent manner (Figure 2B), especially by emodin at the concentration of 15 μM. Therefore, emodin at the concentration of 15 μM was used for following experiments. After emodin treatment, the upregulation of p53 and p21, as well as the downregulation of cyclin D1 were repressed (P<0.001, Figure 2C). In addition, cell apoptosis was inhibited by emodin (P<0.01, Figure 2D). Furthermore, the ratio of cleaved-/pro-caspases-3 was decreased by 0.4 times and the ratio of cleaved-/pro-caspases-9 decreased by 0.38 times (both P<0.001, Figure 2E and F) confirming the result in Figure 2D. These results suggested that hypoxia-induced injuries were inhibited by emodin.

### Emodin up-regulated miR-138 expression

**Figure 3:**
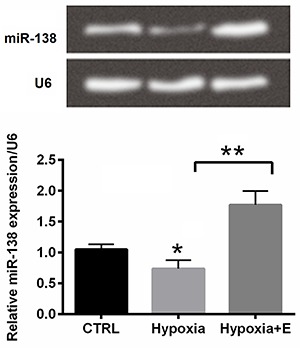
Relative expression of miR-138 was inhibited by hypoxia treatment but then increased by emodin (E) treatment detected by quantitative real-time polymerase chain reaction. Data are reported as means±SD of triplicates. Each experiment was performed three times. *P<0.05, **P<0.001 compared to control or as indicated (one-way analysis of variance).

The possible mechanism of action and the genetic molecule involved in cytoprotection of emodin were explored. miR-138 was chosen to be investigated according to its close correlation with cardiac cell fate (16). Our result in Figure 3 revealed that miR-138 expression was down-regulated 0.7 times in H9c2 cells by hypoxia treatment but further up-regulated 2.4 times by emodin administration. The data implied that miR-138 might be involved in the hypoxia-induced injury of cells and regulate the effect of emodin on hypoxia-induced injury.

### miR-138 overexpression alleviated hypoxia-induced cell injury

**Figure 4:**
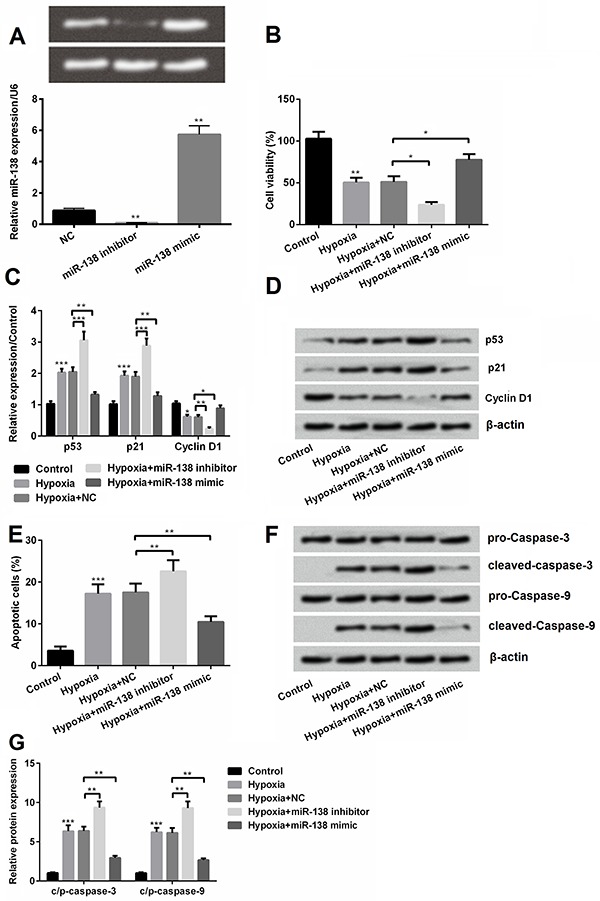
miR-138 overexpression alleviated hypoxia-induced cell injury. *A*, miR-138 expression was up-regulated or down-regulated after transfection assay analyzed by qRT-PCR. *B*, miR-138 overexpression up-regulated in hypoxia-treated cells compared with negative control (NC). *C* and *D*, The protein expressions of p53 and p21 were up-regulated and cyclin D1 was down-regulated by miR-138 overexpression compared with NC. *E* and *F,* miR-138 overexpression decreased cell apoptosis and the effects on apoptosis-related proteins were detected by western blot. Data are reported as means±SD of triplicates. Each experiment was performed three times. *P<0.05, **P<0.01, ***P<0.001 compared to control or as indicated (one-way analysis of variance).

In order to clarify the functions of miR-138 in hypoxia-stimulated H9c2 cells, miR-138 transfections were carried out. miR-138 expression level was altered by transfection with its corresponding mimic and inhibitor. The relative level of miR-138 was significantly reduced 0.1 times and increased 6.5 times after transfection with miR-138 inhibitor and mimic, respectively (P<0.05, P<0.001, Figure 4A). Further experiments were performed and showed that miR-138 overexpression increased cell viability (P<0.05, Figure 4B) and decreased cell apoptosis (P<0.01, Figure 4E) in hypoxia-treated cells compared with NC. Meanwhile, the proliferation-related proteins accumulating level shown in Figure 4C and D revealed that miR-138 overexpression significantly up-regulated p53 and p21 accumulation and downregulated cyclin D1 accumulation, indicating that miR-138 had a positive influence on cell proliferation in hypoxia-treated cells. Similarly, western blot results showed that apoptosis-related proteins, cleaved-caspase 3, and cleaved-caspase-9 were both downregulated by miR-138 overexpression compared with NC in hypoxia-treated cells (Figure 4F-G). On the other hand, miR-138 downregulation led to the opposite results. Taken together, miR-138 overexpression alleviated hypoxia-treated H9c2 cell injury.

### Emodin improved hypoxia injury in H9c2 cells by up-regulating miR-138

**Figure 5:**
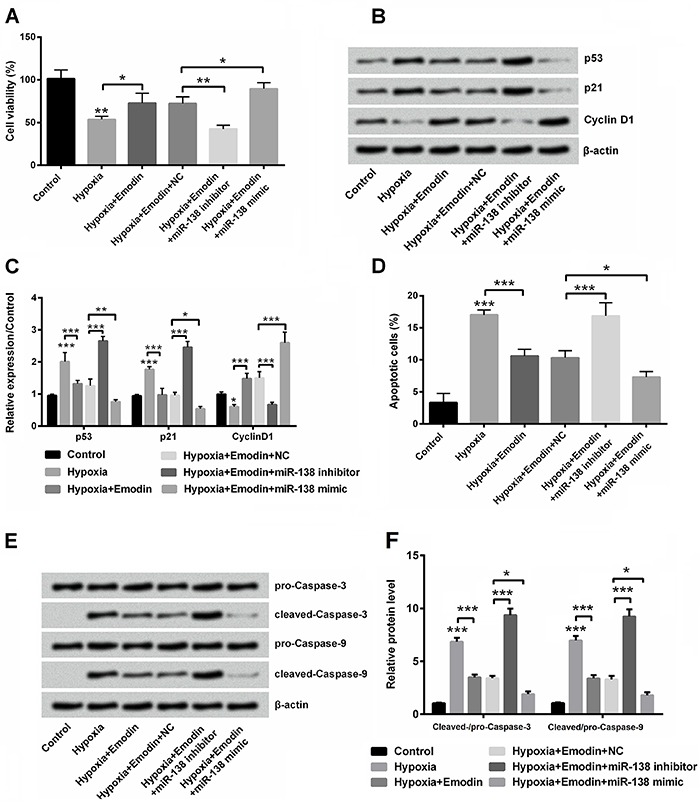
miR-138 modulated the effects of emodin on hypoxia injury in H9c2 cells. *A*, miR-138 positively regulated viability of emodin-treated cells detected by CCK-8 assay. *B*, miR-138 was involved in the effects of emodin on proliferation-related protein expression. *C*, miR-138 negatively regulated apoptosis of emodin-treated cells detected by flow cytometry. *D*–*F*, miR-138 was involved in the effects of emodin on apoptosis-related protein expression. The expression of protein was measured by western blot. Data are reported as means±SD of triplicates. Each experiment was performed three times. NC: negative control. *P<0.05, **P<0.01, ***P<0.001 compared to control or as indicated (one-way analysis of variance).

Knockdown of miR-138 induced a decrease in cell viability although cells were treated with emodin in hypoxia-stimulated cells (P<0.01), but miR-138 overexpression further enhanced the viability-promoting effect of emodin (P<0.05, Figure 5A). Knockdown of miR-138 increased synthesis of p53 2.1 times and p21 by 2.5 times but decreased synthesis of cyclin D1 0.44 times, whereas up-regulating miR-138 expression exhibited the contrary effects (Figure 5B and C). miR-138 silence impaired the apoptosis-inhibitory effect of emodin while miR-138 overexpression promoted the apoptosis-inhibitory effect of emodin on hypoxia-induced cell injury (P<0.05, Figure 5D). Furthermore, the accumulated level of cleaved-caspase-3 and cleaved-casapase-9 was up-regulated by miR-138 inhibitor (P<0.001) and decreased by miR-138 overexpression (both P<0.05, Figure 5E and F). The results indicated that emodin might ameliorate hypoxia injury in H9c2 cells by up-regulating miR-138 expression.

### Emodin activated Sirt1/AKT and Wnt/β-catenin signaling pathways by up-regulating miR-138

**Figure 6:**
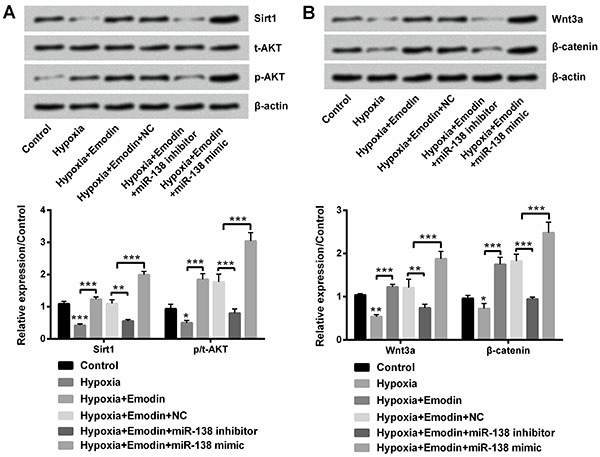
Sirt1/AKT and Wnt/β-catenin signaling pathways were activated by emodin. Emodin promoted activations of (*A*) Sirt1/AKT pathway and (*B*) Wnt/β-catenin pathway by up-regulating miR-138. The expression of protein was measured by western blot. Data are reported as means±SD of triplicates. Each experiment was performed three times. NC: negative control. *P<0.05, **P<0.01, ***P<0.001 compared to control or as indicated (one-way analysis of variance).

The underlying mechanism of miR-138 in the function of emodin was investigated by western blot. Two pathways, Sirt1/AKT and Wnt/β-catenin, were found to be related with MI (22,23). Hypoxia treatment decreased phosphorylation of Sirt1 (P<0.001) and p-AKT (P<0.05, Figure 6A). Besides, hypoxia treatment diminished the accumulated level of Wnt3a (P<0.01) and β-catenin (P<0.05, Figure 6B). However, emodin treatment activated Sirt1/AKT and Wnt/β-catenin pathways by increasing accumulated level of Sirt1, p-AKT, Wnt3a, and β-catenin (all P<0.001, Figure 6A and B). Interestingly, the two pathways were inactivated and promoted in emodin-treated cells when miR-138 was knocked down and overexpressed, respectively (Figure 6). We speculated that emodin activated Sirt1/AKT and Wnt/β-catenin signaling pathways possibly by up-regulating miR-138.

### Emodin activated Sirt1/AKT and Wnt/β-catenin signaling pathways by down-regulating MLK3

**Figure 7:**
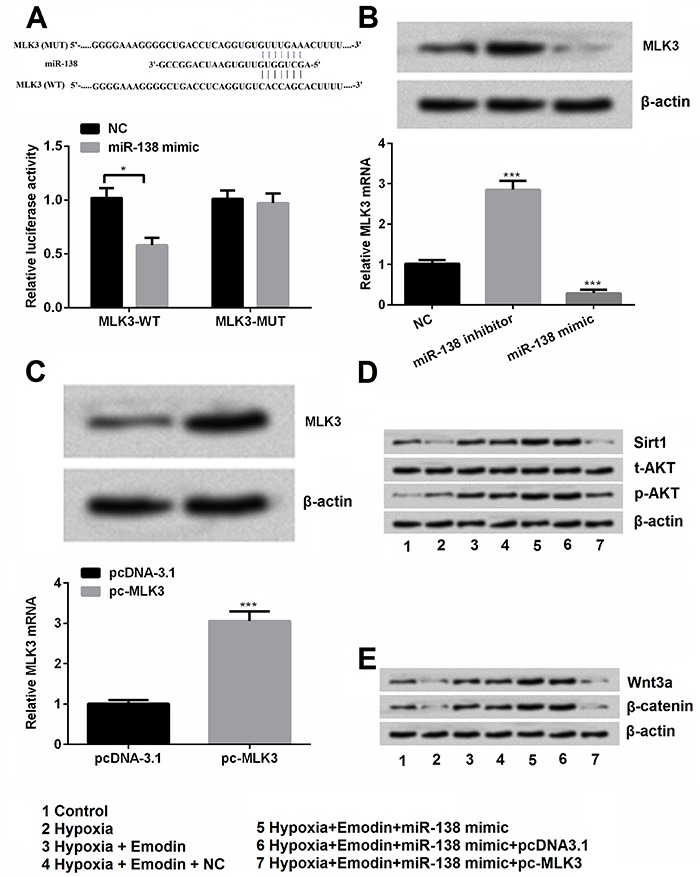
Emodin alleviated hypoxia-induced cell injury via downregulation of MLK3. *A*, Dual luciferase activity assay was performed to confirm miR-138 target MLK3. *B*, miR-138 negatively regulated expression of MLK3. *C*, Overexpression of MLK3 was accessed by transfection with pc-MLK3. Under emodin treatment, (*D*) Sirt1/AKT pathway and (*E*) Wnt/β-catenin pathway were inactivated by co-transfection with miR-138 mimic and pc-MLK3 compared with negative control (NC). The expression of protein was measured by western blot. Data are reported as means±SD of triplicates. Each experiment was performed three times. *P<0.05, ***P<0.001, Student's *t*-test and one-way analysis of variance.

As shown in the luciferase results, MLK3 was proven to be a target of miR-138 (Figure 7A). Further results from Figure 7B demonstrated that MLK3 was negatively regulated by miR-138 overexpression (P<0.001). In order to identify the functions of MLK3 in hypoxia-treated H9c2 cells, pc-MLK3 was transfected. Upregulation of MLK3 by transfection with pc-MLK3 (P<0.001) indicated the high transfection efficiency (Figure 7C). Co-transfection with miR-138 mimic and pc-MLK3 downregulated the accumulated level of Sirt1, p-AKT, Wnt3a, and β-catenin (Figure 7D and E) compared with transfection with miR-138 mimic and pcDNA3.1 in hypoxia-treated cells. These results demonstrated that overexpression of MLK3 inactivated Sirt1/AKT and Wnt/β-catenin signaling pathways, and emodin activated Sirt1/AKT and Wnt/β-catenin signaling pathways by down-regulating MLK3.

## Discussion

Traditionally, emodin has been used as an active ingredient for many Chinese herbal laxatives; however, currently, significant development has been made in investigating its biological effects at cellular and molecular levels and it is emerging as an important therapeutic agent in various fields (24). The present study demonstrated that emodin inhibited hypoxia-induced injury in H9c2 cells via up-regulation of miR-138 as well as activating Sirt1/AKT and Wnt/β-catenin pathways. Our study might provide a new perspective of the modulatory effects of emodin on hypoxia-induced injury in MI, which may widen the research scope of emodin and have the important implication in its future clinical use.

H9c2 cells stimulated with hypoxia exhibited a decreased viability and increased apoptosis, indicating that cell injury was successfully induced. However, emodin treatment increased viability and decreased apoptosis, alleviating the hypoxia-induced injury.

p53, p21, and cyclin D1 are cell cycle-associated proteins (25,26). p53 exerts anti-proliferative effects in response to various types of stress (27). p21 is under the transcriptional control of p53 gene and is a universal inhibitor of cyclin kinases (25). Cyclin D1, a well-known nuclear protein, has been implicated in cell cycle control (28). According to our data, emodin attenuated the up-regulation of p53 and p21, as well as the down-regulation of cyclin D1 in hypoxia-treated cells, which indicated that emodin increased cell proliferation. This result indicated that emodin protected H9c2 cells from hypoxia-induced injury. In addition, the important mediators of apoptosis, caspase-3 and caspase-9, were activated after hypoxia but were down-regulated by emodin treatment.

A recent study demonstrated that emodin protects hyperglycemia-induced injury in PC-12 cells by up-regulating miR-9. Meanwhile, these observations were coupled with the down-regulation of p21, p16, Bax, cleaved-caspase-3 and -9, and the up-regulation of cyclin D1 and Bcl-2 (29), which was consistent with our study. Furthermore, emodin attenuated LPS- and hypoxia/reoxygenation-induced barrier dysfunction in intestinal epithelial (8), which confirmed the hypoxia injury-inhibitory effects of emodin. Taken together, emodin revealed cytoprotective effects, which are consistent with previous studies.

Emodin was reported to protect cells by regulation of miRNAs expression including miR-34a (13), miR-126 (12), and miR-30a-5p (30). Simultaneously, MI was also closely related with miRNAs, such as miR-1 (31) and miR-21 (32). Among these miRNAs, the role of miR-138 in cardiocytes was reported in recent studies. These studies showed that up-regulating miR-138 inhibited hypoxia-induced cardiomyocyte apoptosis (15). Another study demonstrated that miR-138 could protect cardiomyocytes from hypoxia-induced apoptosis (33). The protective effect of miR-138 against cerebral ischemia/reperfusion injury in rats was displayed (34). In our study, miR-138 expression was significantly down-regulated by hypoxia and up-regulated by emodin in H9c2 cells. This result was consistent with previous studies in which hypoxia reduced the expression of miR-138 (15,35). However, this result is contrary with the result from He et al. (33) in which hypoxia increased the expression of miR-138. In the research by He et al. (33), the authors did serum-free starving treatment before the treatment of hypoxia, which induced their cells into G0 phase, while in our experiment, we did not do the starvation treatment, which kept our cells in S phase. The expression of miRNAs is affected by different cell phases (36).

Furthermore, our results demonstrated protective functions of miR-138, which were consistent with the studies that miR-138 protected against hypoxia-induced cell injury (15,33). miR-138 silence impaired the protective effect of emodin against hypoxia-induced injury but miR-138 overexpression enhanced the protective effect of emodin, which indicated that emodin showed the protective effects through up-regulation of miR-138. This result was consistent with a previous study that showed that miR-138 might be able to modulate efficacy of emodin in hypoxia-treated H9c2 cells (11).

Nicotinamide adenosine dinucleotide (NAD)-dependent deacetylase Sirt1 is a type of NAD-dependent deacetylase and has proven to play important roles in multiple biological functions, particularly anti-apoptosis (37). Sirt1 overexpression could promote cell viability and decrease apoptosis of hypoxia-treated osteoblast cells. In addition, Sirt1 activated anti-apoptotic effects by decreasing the activity of caspase-3, -9, and subsequent pathways (38). In our study, under emodin treatment, Sirt1/AKT signaling pathway was activated by miR-138 overexpression in hypoxia-treated cells and the activation of Sirt1/AKT pathway might partly explain the anti-apoptotic property of emodin. Wnt/β-catenin pathway was also activated by emodin in this study. Wnt/β-catenin pathway was shown to promote hepatocyte survival *in vivo* under hypoxic conditions (39), which was consistent with our study.

MLK3 was reported to co-work with miR-138 in hypoxia-induced injury cells (33). In our study, we found that MLK3 was negatively modulated by miR-138 expression. In addition, MLK3 was a target of miR-138, and miR-138 could inhibit the expression of MLK3 and further influence the synthesis of MLK3 protein, and then affect some biological functions in cells. With miR-138 knockdown, the effects on MLK3 were alleviated, causing MLK3 overexpression in H9c2 cells, which inactivated the Sirt1/AKT and Wnt/β-catenin pathways. This cascade reaction might be an explanation about how miR-138 affected cell functions.

The results from the present study demonstrated that emodin might have a protective function in hypoxia-induced injury in H9c2 cells. Emodin promoted cell viability and inhibited cell apoptosis induced by hypoxia possibly by up-regulating miR-138, during which the activations of Sirt1/AKT and Wnt/β-catenin pathways were essential. Emodin and miR-138 intervention may potentially aid in the treatment of ischemic heart disease.
